# Placenta Accreta Spectrum Prophylactic Therapy for Hyperfibrinolysis with Tranexamic Acid

**DOI:** 10.3390/jcm13010135

**Published:** 2023-12-26

**Authors:** Tiyasha Hosne Ayub, Brigitte Strizek, Bernd Poetzsch, Philipp Kosian, Ulrich Gembruch, Waltraut M. Merz

**Affiliations:** 1 Department of Obstetrics and Prenatal Medicine, University Hospital Bonn, Venusberg Campus 1, 53127 Bonn, Germany; 2 Institute for Experimental Hematology and Transfusion Medicine, University Hospital Bonn, Venusberg Campus 1, 53127 Bonn, Germany

**Keywords:** placenta accreta spectrum, expectant management, hyperfibrinolysis, disseminated intravascular coagulopathy, tranexamic acid, fibrinogen, D-dimer

## Abstract

Background: To report on prophylactic therapy for hyperfibrinolysis with tranexamic acid (TXA) during expectant management (EM) in the placenta accreta spectrum (PAS). Methods: This is a monocentric retrospective study of women with PAS presenting at our hospital between 2005 and 2021. All data were retrospectively collected through the departmental database. Results: 35 patients with PAS were included. EM was planned in 25 patients prior to delivery. Complete absorption of the retained placenta was seen in two patients (8%). Curettage was performed in 14 patients (56%). A hysterectomy (HE) was needed in seven (28%) patients; 18 patients (72%) underwent uterus-preserving treatment without severe complications. The mean duration of EM was 107 days. The mean day of onset of hyperfibrinolysis and beginning of TXA treatment was day 45. The mean nadir of fibrinogen level before TXA was 242.4 mg/dL, with a mean drop of 29.7% in fibrinogen level. Conclusions: Our data support EM as a safe treatment option in PAS. Hyperfibrinolysis can be a cause of hemorrhage during EM and can be treated with TXA. To our knowledge, this is the first cohort of patients with EM of PAS in whom coagulation monitoring and use of TXA have been shown to successfully treat hyperfibrinolysis.

## 1. Introduction

Placenta accreta spectrum (PAS) is associated with high maternal morbidity [1,2,3,4]. The rising incidence is mainly attributed to increasing cesarean section (CS) rates [5,6,7,8,9,10]. Retained placenta after vaginal delivery and placenta previa are other risk factors [11,12,13,14]. Forcibly removing an invasive placenta may lead to massive obstetric hemorrhage and a life-threatening coagulopathy. Therefore, cesarean hysterectomy (CS-HE) is considered the gold standard of treatment [15,16,17,18,19]. However, surgical complications should not be disregarded, especially in placenta percreta, when adjacent organs like the bladder are infiltrated and bladder or ureter injuries with associated long-term complications like vesicouterine fistula can be caused [2]. Furthermore, hypervascularization via additional blood supply, mainly from the external iliac arteries, increases the risks of major blood loss even in experienced hands [16,17,18]. Partial excision of invasive placental areas, another treatment option, is also associated with high blood loss. Additional procedures like embolization, vessel ligation, or temporal internal iliac balloon occlusion can reduce blood loss, but there are no randomized controlled trials comparing these procedures [2,19].

Expectant management (EM), leaving the placenta in situ after the delivery of the fetus without any manipulation of the placenta, is associated with a more than 50% reduction in blood loss and need for transfusions [2,20]. A favorable outcome has been reported in up to 85% of cases, avoiding hysterectomy in 19 to 60% of cases [21,22,23,24,25,26,27,28,29,30]. Delayed secondary hysterectomy may be required in 58% within 9 months after delivery [2,25]. Indications include infections and hemorrhage, which may trigger disseminated intravascular coagulopathy (DIC) [23,31,32]. In contrast, hyperfibrinolysis is induced by a massive release of plasminogen activators resulting in increased plasmin concentration and proteolysis of fibrinogen/fibrin. Tranexamic acid (TXA), a synthetic antifibrinolytic agent, inhibits the conversion of plasminogen into plasmin, thus inhibiting hyperfibrinolysis, and is approved for the treatment of hyperfibrinolytic bleeding complications [33]. The uterus and placenta are both rich in plasminogen activators promoting hyperfibrinolysis [31]. Therefore, hemorrhage in PAS may be the result of primary hyperfibrinolysis and may occur without inflammation.

We aimed to report on the prevention and management of hyperfibrinolysis with TXA during EM of PAS.

## 2. Materials and Methods

**Figure 1:**
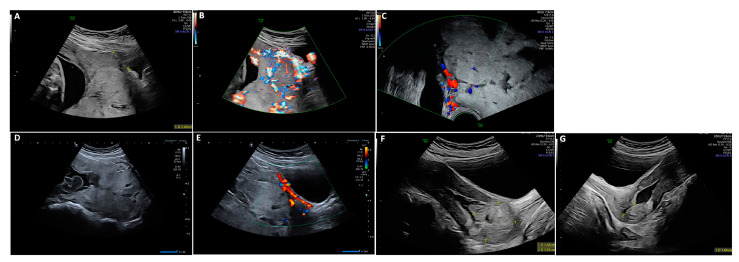
(**A**–**C**) PAS in 34 + 0 weeks of gestation with loss of decidua, bladder wall interruption, placental bulge, and exophytic mass hypervascularity. (**D**,**E**) Placenta in situ on day 13 after CS with persistent bladder wall interruption. (**F**) Placenta in situ on day 63 after CS with a diameter of about 3.5 cm, well separated from the uterine wall, without perfusion. (**G**) Day 122 after CS; fluid-filled uterine cavity surrounded by a hyperechogenic rim.

All patients with PAS presenting at our center between 2005 and 2021 were included. The outcome of 19 patients has already been published, focusing on physical, mental, and reproductive sequelae after the treatment of PAS [31,34]. Antenatal diagnosis of PAS was made using 2D ultrasound, color Doppler, and transvaginal ultrasound [35,36]. Ultrasound criteria included loss of the clear zone or decidua, partial or complete absence of the myometrium layer, sub-placental lacunae, a sudden break-off in the outline of the calcifications characteristic for third-trimester placental basal plates, increased vascular perfusion between the uteroplacental interface reaching the uterine serosa, bladder wall interruption, placental bulge, and exophytic mass hypervascularity (Figure 1) [8,35,36,37,38].

**Figure 2:**
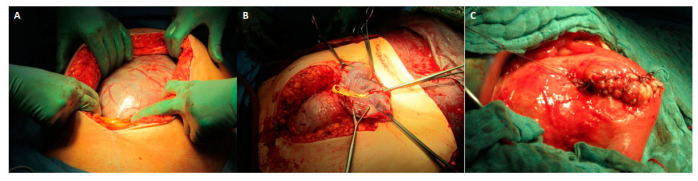
(**A**): A large area of PAS is seen on the front wall of the uterus after abdominal access by a supraumbilical midline incision. (**B**) Uterine fundal longitudinal incision on the back wall of the uterus avoiding the placenta. (**C**) Closed uterotomy after leaving the placenta in situ.

Treatment options (leaving the placenta in situ, partial excision of invasive placental areas, and CS-HE) were discussed in detail with the patients before making an informed decision about further management. Surgery was performed by a team of senior obstetricians and anesthetists with neonatologic stand-by, mainly under general anesthesia. The placenta was avoided by a uterine fundal transverse or longitudinal incision after abdominal access by supraumbilical or lower midline incision (Figure 2) [39]. An intraoperative ultrasound was performed at the surgeon’s discretion to locate the placenta. Uterotonic agents were not administered [40].

Once we observed a decrease in the fibrinogen level and a corresponding increase in the D-dimer level, we started with TXA therapy with an initial dose of 1 g t.i.d. The changes in the concentrations of fibrinogen and the D-dimer were calculated in percent. Completion of EM was defined by the absence of any remaining placental tissue, either by complete absorption, curettage, or HE. Additionally, successful EM was defined as uterus preservation. On the other hand, a failed EM was defined as loss of the uterus. The outcome of hyperfibrinolysis management with TXA therapy was defined as normalization of coagulation screening and prevention or cessation of vaginal bleeding.

Maternal and fetal outcomes, laboratory and ultrasound reports, and follow-ups were retrospectively collected. The Institutional Review Board of the University of Bonn Medical Faculty does not require formal approval for retrospective observational studies. Only basic statistical tests were performed. This included the calculation of the mean value and plotting the values in a figure using Microsoft Office Professional Plus 2016 (Microsoft Corp., Redmond, WA, USA).

## 3. Results

**Figure 3:**
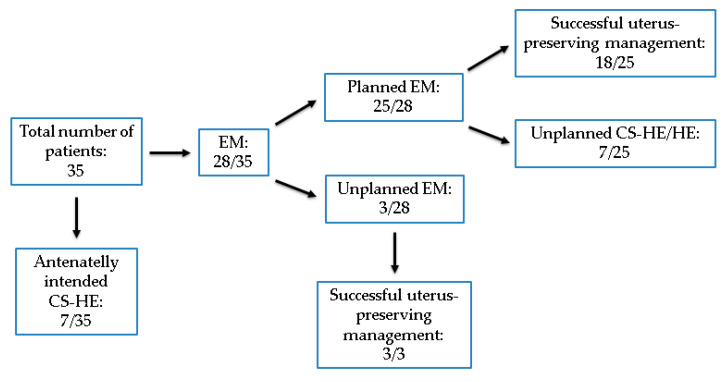
Placenta accreta spectrum: management and outcome. EM—expectant management; CS—cesarean section; HE—hysterectomy.

During the study period, thirty-five patients with PAS were included. The diagnosis was established antenatally in 32 (91.4%) patients. A large area of PAS was detected during delivery in three patients. There was no maternal death. Details of the study population are listed in Table 1 and Figure 3. The mean week of gestation at first diagnosis was 28 + 4 weeks, and at delivery, it was 35 + 0 weeks. Thirty (85.7%) patients had at least one previous CS, and eleven (31.4%) patients had at least one curettage. Seven (20%) patients had a history of both CS and curettage. Placenta previa totalis was diagnosed in 24 (68.6%) patients. Two (5.7%) patients reported a history of PAS. Five (14.3%) patients had no prior CS, and one of these suffered PAS in her first pregnancy with smoking as a sole risk factor. Uterine malformations such as uterus bicornis were seen in three (8.6%) patients. Placenta membranacea was present in four (11.4%) patients, of whom two had large parts of scattered ingrown placenta on the front and back walls of the uterus. Scheduled elective CS-HE was performed in seven (20%) patients, with the latest in 2017. Two (5.7%) patients were delivered by emergency CS in the 28th week of gestation due to vaginal hemorrhage. An elective CS-HE due to completed family planning was chosen by five (14.3%) patients.

### 3.1. Planned EM, Failed

EM was the intended approach for the treatment of PAS in 25 (89.3%) patients after 2007 (Table 1 and Table 2). In two (8%) patients, partial placental detachment occurred during CS. Complete removal of the placenta was achieved in both cases. In the first patient, hemostatic treatment consisted of the insertion of a chitosan-coated gauze (Celox^®^, Crewe, UK) and a Bakri balloon. The estimated blood loss (EBL) was 4.000 mL. The patient received an intravenous bolus injection of 1 g TXA, five units of packed red cells (PRC), and four units of fresh frozen plasma (FFP). The second patient, who presented with placenta increta and membranacea required compression sutures of the placental bed, insertion of tabotamp, and chitosan-coated gauze (Celox^®^) for control of hemorrhage. EBL was 3.000 mL, with a transfusion of three units of PRC and four units of FFP.

### 3.2. Planned EM, Successful

**Figure 4:**
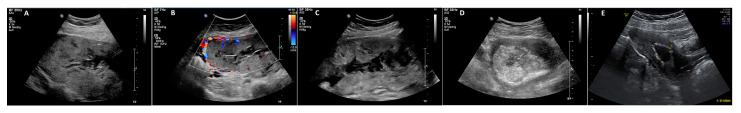
(**A**) Placenta in situ on day 9 after CS. (**B**) Placenta in situ on day 15 after CS. (**C**) Regressively altered placenta in situ on day 64 after CS. (**D**) Placenta in situ well separated from the uterine wall without perfusion on day 106 after CS. (**E**) Day 194 after CS with a hyperechogenic rim around a fluid-filled uterine cavity.

In total, two (8%) of the twenty-five patients with intended EM showed complete absorption of the retained placenta (Figure 1 and Figure 4). Fourteen patients (56%) underwent secondary curettage with complete removal of the placenta. Four (28.5%) patients received units of PRC during curettage (two, four, and eleven units). One patient presented with fever and vaginal bleeding and needed an early discontinuation of therapy due to bleeding on day 18 after CS (Table 2). An emergency curettage was performed with the insertion of a chitosan-coated gauze (Celox^®^). The following day, a second curettage was performed due to bleeding. This time, a Bakri balloon was inserted into the uterus. The patient received nine units of PRC and six FFP in total. Secondary HE was needed in seven patients (28%): one during CS due to severe bleeding, one due to massive intravesical bleeding because of suspected invasion of the bladder wall on day 8 after CS, one after failed placental removal during curettage on day 52 after CS, and two due to infection and perforation during curettage on days 56 and 86 after CS, respectively. Planned secondary HEs were performed in two (8%) patients, one due to a residual large area of placenta previa et increta near the cervix on day 121 after CS, and the other due to completed family planning on day 59 after CS (Table 2 and Table 3). Thus, 18 patients (72%) were successfully treated with uterus-preserving methods; in 15 (93.7%) patients, this was without severe complications (Table 1).

### 3.3. Uplanned EM, Successful

In three (10.7%) patients, the diagnosis was established during delivery, and parts of abnormally invasive tissue were left in utero (Table 2). Of these, two patients delivered vaginally and one via CS. All three were successfully treated with complete absorption of the residual placenta. The first patient had a curettage for incomplete placenta after operative vaginal term delivery. An abnormally invasive placental area of approximately 7 × 6 cm on the uterine fundus was detected, and it was left in situ due to stable bleeding and proper uterus contraction. EM was followed, with spontaneous tissue loss on day 70 after delivery. The second patient had a curettage for retention of the placenta after full-term vaginal delivery. An abnormally invasive placental area in the uterine fundus was found. This patient was additionally treated with methotrexate. The increased part of the placenta was completely absorbed after 153 days, and she became pregnant again one year later. The outcome of the following pregnancy was a secondary CS in the 30 + 2 weeks of gestation due to contractions after a preterm premature rupture of membranes in the 22 + 6 weeks of gestation. The placenta was adherent but could be removed completely. The EBL was 700 mL. The third patient had an elective CS at term for breech presentation. During surgery, the abnormally invasive placental area was left in situ. Fifteen u-shape and three B-Lynch sutures were placed for hemostatic control. EBL was 1.800 mL, and she did not require units of PRC. The follow-up of the patient was conducted at 589 days. The patient had a very thin myometrium on the back wall of the uterus. Curettage was assessed to be high risk and was, therefore, not performed. The placenta was eventually completely absorbed.

### 3.4. Follow-Up during EM (n = 25)

The mean duration of EM was 107 days (range 8–589 days, Table 2). All patients were monitored weekly for six to eight weeks after delivery. Thereafter, monitoring was extended to monthly check-ups. Each visit included a clinical examination, ultrasound, and laboratory investigations (ß-hCG, PAPP-A, CRP, full blood count, fibrinogen, D-dimer, prothrombin time, activated partial thromboplastin time, and antithrombin). ß-hCG decreased steadily and became negative before cessation of blood flow in the retained placenta [2]. Prothrombin time, activated partial thromboplastin time, and antithrombin concentration remained within normal ranges. In case of abnormal findings, surveillance was intensified. Eleven patients (44%) required readmission for febrile complications, including one case of coxitis, which occurred two weeks after unplanned HE 86 days after CS (Table 2). Coxitis was assumed to be a complication of bacteremia and was treated by arthrotomy and lavage; for details see Table 2.

### 3.5. Hyperfibrinolysis Management

Hyperfibrinolysis, which is defined as a combination of decreased fibrinogen and elevated D-dimer levels, was detected in 11 out of 25 (44%) patients. The platelet count was normal in all these patients. The outcome of these patients is summarized in Table 3.

**Figure 5:**
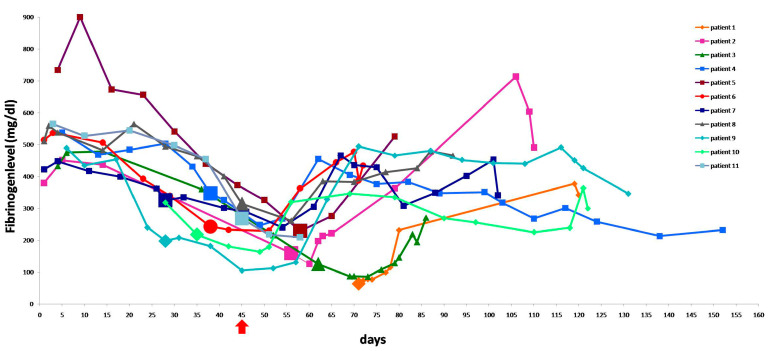
Fibrinogen during EM and treatment with TXA in 11 patients. The days when TXA therapy was started are highlighted in bold. The mean day of initiating TXA therapy after CS was day 45 (arrow).

**Figure 6:**
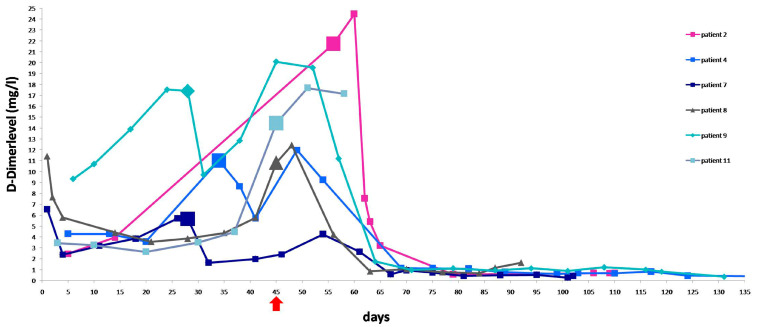
D-dimer during EM and treatment with TXA in six patients. The days when TXA therapy was started are highlighted in bold. The mean day of initiating TXA acid therapy after CS was day 45 (arrow).

The mean day of onset of hyperfibrinolysis and initiation of treatment with TXA was post-operative day 45 (range 28–71). The mean duration of EM in this subgroup was 101 days (range 59–152) under continued TXA medication. The mean fibrinogen nadir before TXA was 242.4 mg/dL (range 64–431 mg/dL, normal range 180–355 mg/dL), with a mean drop of 29.7% in fibrinogen levels (Figure 5). D-dimer levels were available for six patients, as shown in Figure 6. The mean D-dimer concentration before initiation of TXA treatment was 13.5 mg/L (range 5.7–21.7 mg/L; normal range: 0–0.5 mg/L), with a mean increase of 273.7%. Our starting dose of TXA was 1 g t.i.d. during TXA therapy, close monitoring was continued to adjust the dose, if necessary. The maximum dose of TXA was 1.5 g t.i.d. Curettage was performed in seven (63.6%) patients (Table 3). One patient required a laparotomy due to a bladder injury. Another patient presented with complete absorption of the placenta. A planned HE was performed on two patients. Emergency HE for intractable bleeding after uterine perforation with a bladder injury during curettage was required in one patient at day 86 post-partum. The patient received 26 units of PRC and 20 units of FFP. Therefore, in this subgroup, eight patients (72.7%) could be treated by uterus-preserving methods. Eight weeks after CS, one patient presented with massive bleeding and not detectable fibrinogen levels. The patient had received TXA therapy but, due to normalized fibrinogen levels, TXA had been stopped one week prior to the bleeding. Curettage could be performed on this patient. The patient needed a transfusion of eleven units of PRC, six units of FFP, two units of platelet concentrates, and a Bakri catheter. However, this patient could not be included in Table 3 and Figure 5 due to a lack of regular measurements of her coagulation profile before curettage. There were no thromboembolic complications (deep venous thrombosis or pulmonary embolism) in any of the patients.

A further follow-up showed that three of our patients conceived again after PAS; one patient among them conceived three times. One patient developed Asherman syndrome, and one suffered from short-term depression. The mean duration of inpatient stay was eight days (range 2–34). One infant died two months after delivery at 27 + 2 weeks of gestation because of vaginal hemorrhage, corresponding to a 2.8% infant mortality rate, and most of the newborns were discharged with their mothers within the first week after delivery.

## 4. Discussion

PAS is a challenging problem worldwide, with a high maternal morbidity [1,2,7]. Although conservative management for PAS is associated with lower surgical morbidity at CS, CS-HE is still the gold standard of treatment [41]. Antenatal diagnosis and centralized management are associated with lower maternal and fetal morbidity [20,42]. Exact placenta localization is mandatory for the determination of the uterine incision.

Advantages of EM include fertility preservation and reduction of surgical complications such as severe hemorrhage [2,20]. Differentiating DIC from hyperfibrinolysis is essential for the correct management [30,31]. Infection is the most likely cause of DIC, and removing the focus of infection surgically is essential to reduce further complications [30]. If secondary hyperfibrinolysis occurs in the context of DIC, TXA treatment will fail to control bleeding. TXA treatment should be considered only in isolated hyperfibrinolysis. Thus, regular evaluation of coagulation and inflammation parameters is recommended in patients with EM. The coagulation screening in our center included weekly measurements of fibrinogen and D-dimer levels. Any changes should be considered an indication for initiation of 1 g TXA t.i.d. In our study, we detected a mean decrease in fibrinogen levels of 29.7% and a concomitant D-dimer increase of 273.7% before the start of TXA. We initiated TXA in patients with a relevant mean drop of 128.7 mg/dL (range 37–278 mg/dL) in fibrinogen even if it was still above the normal range (355 mg/dL) for non-pregnant patients. Close monitoring should be continued under TXA treatment to adjust the dose. In case of insufficient increase of the fibrinogen level, continuous decrease in the level, or vaginal bleeding under TXA therapy, the dose of TXA was increased up to 1.5 g t.i.d. Prothrombin time, activated partial thromboplastin time, and antithrombin were not relevant parameters for monitoring in our cohort [31]. Coagulation screening should include platelet count to exclude DIC. All our patients treated with TXA had normal platelet counts.

Close monitoring during EM is recommended, with weekly follow-ups in the first eight weeks after delivery, with special attention to coagulation parameters in the second month after delivery (Table 4) [43]. If the patient is stable, monitoring intervals can be extended to monthly from the third month. Every check-up should include a clinical examination for bleeding, abdominal pain, and temperature, as well as an ultrasound with measurement of the size and perfusion of the residual placenta and assessment of fluid retention as a sign of partial detachment of the retained placenta. Furthermore, laboratory tests should include infection parameters, blood count, and ß-hCG in addition to coagulation screening [2].

Hyperfibrinolysis did not occur in the first four weeks after CS in our study. The mean beginning of TXA therapy was on day 45 after CS. Fibrinogen levels can normalize under TXA therapy, but treatment should be continued in our opinion. Severe bleeding in one of our patients eight weeks after CS might have been prevented if TXA had been continued.

Patient management may be difficult due to the long duration and frequent check-ups required with EM (up to six months) [2]. This information needs to be included in the initial counseling. The patient should be briefed about symptoms like abdominal pain, bad smell of vaginal discharge, risk of infection, bleeding, and dysuria. Any decision about further treatment should be made jointly. In one of our previous publications, we discovered that 50% of women after HE reported difficulties in accepting the loss of fertility. All women with prenatally diagnosed high-grade invasive disease were satisfied with their choice of treatment [34].

## 5. Conclusions

In conclusion, although CS-HE is still the gold standard for treatment of PAS, EM of PAS is a safe treatment option with the intention of reduced blood loss at the time of delivery and reduced perioperative complications if delayed hysterectomy is performed [2,20,25]. Close clinical and laboratory follow-up is mandatory, with special attention to early signs of hyperfibrinolysis, which usually appear four to six weeks after CS. TXA can safely be used to treat hyperfibrinolysis and prevent severe bleeding complications and should be continued even after normalized coagulation screening.

In our retrospective cohort, we hypothesize that the use of TXA contributed to successful EM of PAS and reduced complications during follow-up. Two-thirds of our patients with planned EM successfully underwent uterus-preserving treatment; in the subgroup of patients treated with TXA, the rate increased to nearly three-quarters. Hyperfibrinolysis occurred in 11 (44%) patients during EM. To our knowledge, this is the first case series of patients with prospective management of PAS in whom coagulation monitoring combined with TXA treatment successfully reverted hyperfibrinolysis. Further details remain to be investigated in prospective studies. This refers, e.g., to fibrinogen cut-off values for evaluating initiation and discontinuation of TXA therapy and dose-finding.

## Figures and Tables

**Table 1:** Patient characteristics.

Total number of patients	35
Maternal age, mean (range)	32.8 (21–41)
Gravidity, mean (range)	3.6 (1–13)
Parity, mean (range)	2.2 (0–11)
Timing of diagnosis, n (%)	
antepartum	32/35 (91.4%)
Intrapartum	3/35 (8.6%)
Type of PAS	
increta	17/35 (48.6%)
percreta	14/35 (40%)
increta/percreta and membranacea	4/35 (11.4%)
Placenta previa in current pregnancy	26/35 (74.3%)
total	24/35 (68.6%)
Partial	2/35 (5.7%)
GA at diagnosis (weeks), mean (range)	28 + 4 (9 + 2–36 + 3)
GA at delivery (weeks), mean (range)	35 + 0 (27 + 2–37 + 1)
Risk factors, n (%) (multiple risk factors possible)	
Previous CS	30/35 (85.7%)
1 CS	18/30 (60%)
2 CS	5/30 (16.7%)
3 CS	3/30 (10%)
>3 CS	4/30 (13.3%)
Number of patients without previous CS	5/35 (14.3%)
Curettages	2/5 (40%)
Placenta previa in current pregnancy	1/5 (20%)
History of placenta increta	1/5 (20%)
Smoking	1/5 (20%)
Curettages (number of curettages)	11(1–5)/35 (31.4%)
Uterus bicornis/arcuatus	3/35 (8.6%)
History of endomyometritis	1/35 (2.9%)
Asherman syndrome	1/35 (2.9%)
History of intrauterine device	1/35 (2.9%)
History of placenta increta	2/35 (5.7%)
Assisted reproduction by IVF/ICSI	2/35 (5.7%)
Smoking	4/35 (11.4%)
**EM**	28/35 (80%)
Planned EM	25/28 (89%)
Intrapartum diagnosis (unplanned), managed with EM	3/28 (11%)
Successful uterus-preserving management	21/35 (60%)
Successful uterus-preserving management in planned EM	18/25 (72%)
Antenataelly intended CS-HE	7/35 (20%)
Unplanned CS-HE/HE	7/25 (28%)

PAS—placenta accreta spectrum; GA—gestational age; CS—cesarean section; IVF—in vitro fertilization; ICSI—intracytoplasmic sperm injection; HE—hysterectomy; EM—expectant management.

**Table 2:** Outcome of cases with EM (n = 28).

**EM n (%)**	**28**
Mean duration of EM (days)	107 (8–589)
**Planned EM**	25/28 (89.3%)
Complete placental absorption	2/25 (8%)
Planned curettage	14/25 (56%)
*Emergency curettage due to bleeding*	1/25 (4%)
Intraoperative placental detachment, complete removal during CS	2/25 (8%)
HE	7/25 (28%)
*HE during CS*	1/25 (4%)
*Unplanned HE*	4/25 (16%)
*Planned HE*	2/25 (8%)
**Unplanned EM** *(one vaginal delivery, one operative vagianal delivery, one CS)*	3/28 (10.7%)
Complete placental absorption	3/3 (100%)
**Complications following planned EM, n (%)** **(multiple possible)**	25
Abnormal coagulation screening	11/25 (44%)
Infection	11/25 (44%)
Abdominal pain	6/25 (24%)
Nausea	1/25 (4%)
Gingival bleeding	1/25 (4%)
Dysuria	1/25 (4%)
Blood transfusions (2–26 units)	11/25 (44%)
During HE (8–26 units)	5/7 (71.4%)
During curettage (2–11 units)	4/14 (28.6%)
During CS (4 units)	2/2 (100%)

CS—cesarean section; HE—hysterectomy; EM—expectant management.

**Table 3:** Characteristics of patients with treatment of hyperfibrinolysis with tranexamic acid.

Pat.	Start of TXA (Day)	Fibrinogen (mg/dL) at Start of TXA (Previous Value), Normal Range 180–355 mg/dL	D-Dimer (mg/L) at Start of TXA (Previous Value), Normal Range: 0–0.5 mg/L	Therapy Outcome	Duration of EM (Days)
**1**	71	64 (ND)	ND	Curettage	119
**2**	56	159 (437)	21.75 (3.9)	Curettage	109
**3**	52	216 (360)	ND	HE due to infection and perforation with injury of bladder during curettage (24 PRC transfusion)	86
**4**	34	431 (503)	11.02 (3.6)	Complete absorption (Figure 1)	152
**5**	58	229 (326)	ND	Curettage	82
**6**	38	243 (339)	ND	Curettage, laparotomy due to perforation and injury of bladder without HE	71
**7**	28	325 (362)	5.66 (3.86)	Curettage (two PRC transfusion)	102
**8**	45	315 (401)	10.83 (5.77)	Curettage	91
**9**	28	198 (240)	17.41 (13.89)	Curettage, but residual placenta	116
**10**	35	218 (318)	ND	Elective HE due to large area of placenta previa et percreta near the cervix	121
**11**	45	268 (454)	14.45 (4.46)	Elective HE due to completed family planning	59
**Mean**	45 (28–71)	242.4 (64–431) mean % drop: 29.7%	13.5 (5.7–21.7) mean % rise: 273.7%		101 (59–152)

TXA—tranexamic acid; Pat.—patient; ND—not determined; PRC—packed red cells; HE—hysterectomy.

**Table 4:** Our monitoring protocol of expectant management of placenta accreta spectrum.

Suggested Follow Up
Weekly follow-up in the first two month, thereafter every two-four weeks
Clinical examination
Ultrasound
Laboratory tests
(for detailed algorithm for coagulation screening we refer to Schröder et al., 2015 [31])

## Data Availability

The datasets used and/or analyzed during the current study are available from the corresponding author upon reasonable request.
